# A streamlined cloning workflow minimising the time-to-strain pipeline for *Pichia pastoris*

**DOI:** 10.1038/s41598-017-16172-0

**Published:** 2017-11-17

**Authors:** Kate E. Royle, Karen Polizzi

**Affiliations:** 1 Department of Life Sciences, Imperial College London, London, UK; 2 Centre for Synthetic Biology and Innovation, Imperial College London, London, UK

## Abstract

Although recent advances in *E. coli* self-assembly have greatly simplified cloning, these have not yet been harnessed for the high-throughput generation of expression strains in the early research and discovery phases of biopharmaceutical production. Here, we have refined the technique and incorporated it into a streamlined workflow for the generation of *Pichia pastoris* expression strains, reducing the timeline by a third and removing the reliance on DNA editing enzymes, which often require troubleshooting and increase costs. We have validated the workflow by cloning 24 human proteins of biopharmaceutical value, either as direct therapeutics or as research targets, which span a continuous range in size and GC content. This includes demonstrating the applicability of the workflow to three-part assemblies for a monoclonal antibody and its single-chain antibody fragments derivatives. This workflow should enable future research into recombinant protein production by *P. pastoris* and a synthetic biology approach to this industrial host.

## Introduction

Biologics have revolutionised healthcare and their production is big business: in the US, total sales exceeded $140 billion for 2013^1^. Although the sector is dominated by the production of monoclonal antibodies from mammalian cells, increasing competition from biosimilars and patent expiration puts pressure on companies to innovate. With this in mind, yeast platforms have great potential in the discovery phases of biopharmaceutical production as they can rapidly generate candidates for preliminary testing.

For decades *Saccharomyces cerevisiae* has been the popular choice for expression of proteins for biotechnology applications; however, the hypermannosylation of glycoproteins reduces its utility for biotherapeutic production. Attempts to glycoengineer the host have been limited as the deletion of *alg3* results in a severe growth retardation phenotype^2^. In contrast, the methylotrophic yeast *Komagataella phaffii* ^3^, commonly referred to as *Pichia pastoris*, has been engineered to produce a humanised glycan profile and successfully commercialised^4^. Additionally, the industrial heritage, along with its GRAS status and capacity for high density culture, makes it an attractive host for biopharmaceutical production. Despite these clear advantages, *P. pastoris* has not been widely adopted, as unlike *S. cerevisiae*, only a few research labs have made efforts to expand the molecular toolbox^5,6^, increase cloning efficiency^7,8^ and time-to-product pipelines^9^.

A typical workflow for heterologous protein expression with *P. pastoris* is as follows: the target is cloned into a vector such as pPICZαA/B/C (ThermoFisher) and enzymatically digested to produce a linear cassette with 5′ and 3′ regions of homology. This subsequently recombines into the *P. pastoris* genome through homologous recombination or non-homologous end joining. Unlike *S. cerevisiae*, the significant activity of the latter has hindered approaches using direct assembly of DNA parts into the genome *in vivo*. A recent study has demonstrated direct assembly of blue fluorescent protein (BFP) into the panARS-based episomal vector *in vivo*, however, to ensure a low background the authors were required to split the resistance marker into two fragments and use a 6-fold excess of the BFP insert^10^. While a valuable proof-of-concept, the applicability to genomic integration to generate a wide range of industrially relevant expression strains has yet to be demonstrated. Therefore the reliance on restriction enzymes in both cloning and linearization for transformation in the typical workflow prevents the development of a standard protocol that could be amenable to high-throughput automation. Furthermore, restriction cloning often introduces scar sequences limiting expression of proteins with native N-termini.

This research addresses these issues by devising a streamlined workflow for the parallel integration of targets into the same expression vector by taking advantage of a recent emerging trend to use *E. coli* to self-assemble parts *in vivo*. Here, the parts to be assembled are (i) amplified with overlapping homology by PCR; and (ii) directly transformed into cells. Despite first being identified in 1993^11,12^, and having numerous published protocols (Table 1), this extraordinarily simple cloning method has not been widely adopted in academia or industry. One reason for this could be the lack of a consensus in protocols, or an incomplete analysis of the limitations. It may be that the construction of complicated, multi-part assemblies do require *in vitro* enzymatic assembly such as the Gibson method^13^, but the majority of single target cloning would benefit from a high-throughput, automatable standard cloning protocol.

**Table 1:** Publications employing *E. coli* self-assembly cloning.

Name/ reference	PCR Enzyme; Cycles	Treatment	bp	Amount of DNA		Transformation
				Vector	Insert	
Bubeck^11^	—	—	33–42	200 ng	200 ng	DH5α^**C**^
IVC^12^	Amplitaq; 30	Pptn.	150	18 ng	50 ng	JC8679^**E**^
Parrish^30^	Herculase; 30	Pptn.	20	∼50 ng	∼50 ng	KC8^**C**^
FastCloning^31^	Phusion, 18	DpnI	15–17	—	1:1	XL-10 Gold^**C**^
Jacobus^32^	Phusion; 30	DpnI; GE	30	100–250 ng	2:1	DH5α^**C**^
Kostylev^15^	PrimeSTAR Max/Q5	DpnI; C&C	50	1–100 ng	5:1	DH5α^**C**^
AQUA^14^	Q5; 20	GE	32	12 ng/kB	3:1	10β/TOP10, 1 hr RT premix,^C^
IVA^33^	Phusion; 18	DpnI	15–20	2 µL combined PCR		XL-10 Gold^**C**^
OEPR*^34^	KOD-FX; 25	DpnI	15	5 µL digested DNA		TOP10F’^**C**^

Where multiple variables were tested, optimal conditions have been reported. Pptn.: Alcohol precipitation; GE: Agarose gel extraction; C&C: PCR reaction clean up; bp: base pairs of homology; ^C^Chemically competent; ^E^Electrocompetent and *combined overlap extension PCR and self-assembly in *E. coli*.

The self-assembly process greatly simplifies cloning and benefits from reduced labour, equipment time, enzymes, molecular biology kits; and, importantly, limits troubleshooting. We have validated it by evaluating the efficiency of the scarless cloning of 24 industrially relevant target proteins from human cDNA (and showed functional expression in selected cases). While this demonstrates a high-throughput and automatable approach for cloning targets into expression vectors with *E. coli*, throughput is still limited by the culturing, extraction, digestion and gel purification of microgram quantities of DNA for transformation into *P. pastoris*. Therefore, we have established a new method that generates sufficient DNA for transformation using colony PCR and redesigned the most widespread expression vector to optimise this process. In total, we have reduced the strain development timeline by a third and developed a method that is automatable at all steps. The newly developed methodology will accelerate the design-build-test cycles that underpin both synthetic biology approaches to engineering biotechnological processes and the increasing focus on quality-by-design in industrial research and development.

## Results

In this work we have exemplified the simple and high-throughput by cloning 24 genes coding for human proteins of biopharmaceutical value; including 19 targets from human cDNA, the broadly neutralising anti-HIV antibody VRC01 and four single-chain antibody fragment (scFv) derivatives of VRC01. While cloning methods using *E. coli* self-assembly have been previously published (Table 1), a thorough analysis of their limitations in terms of locations of regions of homology, insert size and GC content have not yet been made, reducing their widespread adoption. These cloning advances have been combined with a new streamlined method for generating *Pichia pastoris* strains reducing the time from five days to three and requiring considerably less labour, reagents and consumables.

### *E. coli* DH5α mediated assembly

Our starting point was two recent publications that take advantage of the low, native recombination propensity in *E. coli* DH5α cells^14,15^. Our aim was to derive a high-throughput method for the parallel integration of single targets into the same expression vector, pPICZαA. Therefore, we designed primers that would amplify under uniform conditions, selected PCR reaction clean-up over gel extraction steps and chemically competent DH5α cells such that the whole process is amenable to automation in 96-well plates.

**Figure 1:**
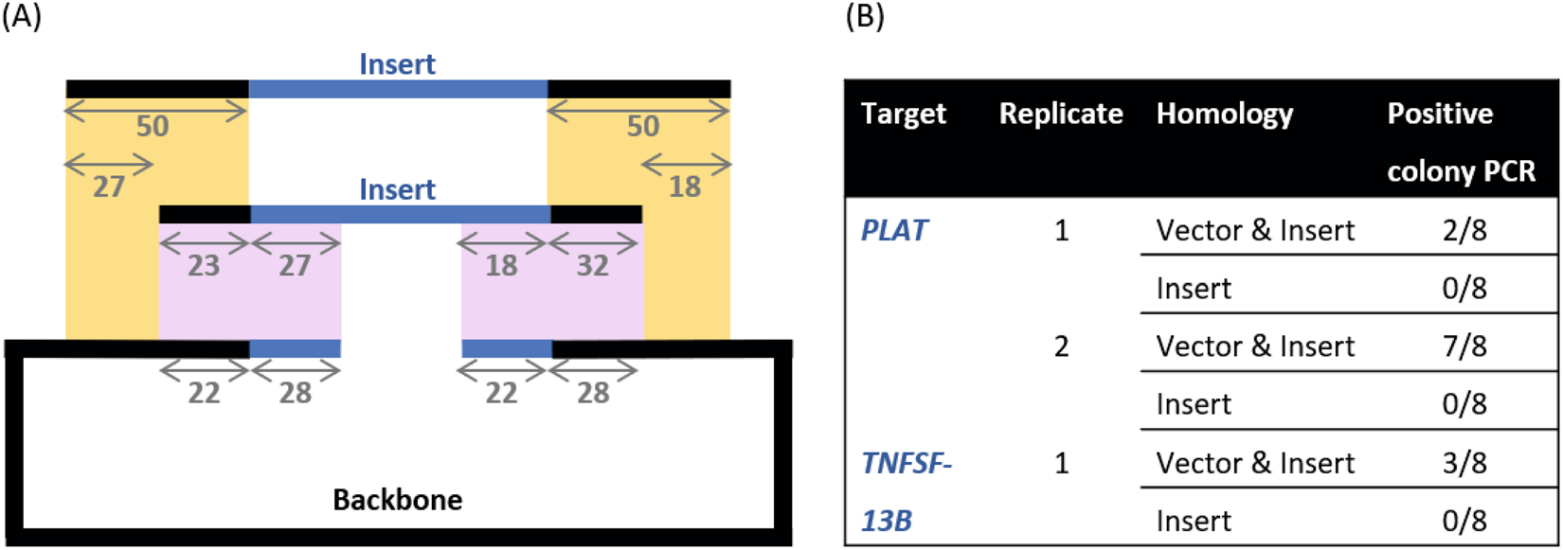
Testing the location of regions of homology. (**A**) Both regions have exactly 50 base pairs of homology, however; in the top scheme this is included entirely on the insert allowing the same vector PCR product to be used for any insert. In the bottom scheme, the homology is divided between the insert and the vector, with the exact split determined by the melting temperature of the amplification site, preventing parallelization. The base pairs for primers to construct pPICZαA[*PLAT*] have been included as an example. (**B**) Colony PCR results from assemblies of tissue plasminogen activator (TPA, *PLAT*) and B lymphocyte stimulator (BLyS, *TNFSF13B*) into pPICZαA.

Our initial tests explored the importance of the location of the region of homology: if backbone amplification was non-specific to the target insert, only one backbone would need to be generated for cloning a range of targets, increasing the throughput and decreasing the primer costs (Fig. 1 (A)). We designed PCR primers to amplify tissue plasminogen activator (TPA, encoded by *PLAT*), a target midrange in size with exactly 50 base pairs of homology either only on the insert or split between the insert and the vector. Transformations produced a minimum of 52 colonies, with those containing homology on just the insert producing between 5 and 10-fold more colonies than those with homology split between the vector and the insert. However, colony PCR screens of eight colonies from the condition where the homology was contained only on the insert amplified the backbone vector (Fig. 1 (B)). As both reactions used the same parental pPICZαA template, this is unlikely to be the result of incomplete DpnI digestion. This suggests the backbone PCR product without homology to the insert can re-circularise in the absence of insert with a high efficiency, even though the PCR amplification removed a 55 base pair section of the multiple cloning site and no homology exists between the ends to mediate re-circularisation. Bioinformatics analysis of the inserts with and without the extra homology, which differ by an extra 27 base pairs at the 5′ terminus and 18 base pairs at the 3′ terminus, did not show any additional hairpin loops or self-complementarity that might explain the difference in assembly efficiency between the two strategies. To ensure the result was not a peculiarity of TPA, the process was repeated with B lymphocyte stimulator (BLyS, encoded by *TNFSF13B*) with comparable results (Fig. 1 (B)). While the data suggests that assembly cannot occur when homology is based solely on the insert, we have previously inserted superfolder GFP into pPICZαA with 50 base pairs of homology on the insert only (data not shown). In that case, only one positive colony was found from a screen of 16, suggesting the process is technically feasible, but inefficient.

### PCR product transformation of *P. pastoris* GS115

The simple, streamlined approach to cloning with *E. coli* prompted us to explore the rest of the process of generating *P. pastoris* expression strains. We wished to develop an alternative to the plasmid-based transformation system, as the use of restriction enzymes prevents standardisation, increases processing time and is resource intensive – requiring 5–20 µg linearised DNA^16^. Therefore, we investigated the possibility of using PCR products directly from colony screening to transform *P. pastoris*, which would combine detection of positive *E. coli* transformants with production of DNA for transformation, increasing the efficiency and removing the overnight steps. Previous research has used overlap-extension PCR to generate cassettes for antibody expression from *P. pastoris* ^9^. While this has clear advantages in modular assembly and obviates *E. coli* transformation, it required eight PCR reactions to generate the fragments, three reactions to join the fragments into three segments, and a final reaction to generate the cassette. As the authors required 5–10 µg DNA for transformation, multiple reactions were precipitated to achieve the DNA concentration required. Here, we wished to test whether a single colony PCR reaction could generate the DNA required for transformation.

**Figure 2:**
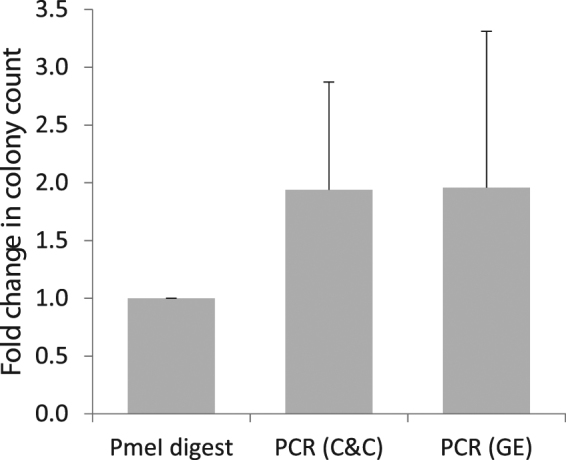
Testing the efficiency of linearised plasmidis DNA versus PCR products using pPICZαA. DNA was prepared by PmeI digest or PCR, where the latter was processed with reaction clean-up (C&C) or gel extraction (GE). For each transformation, 198.6 ng DNA was transformed into electrocompetent GS115 cells from the same batch. Colonies were counted and the results expressed as a fold change in colony count, compared to the PmeI digest. Error bars indicate the standard deviation in four biological repeats, where both separate DNA and batches of electrocompetent cells were prepared.

One caveat is that plasmid digestion samples usually undergo gel extraction, in contrast to PCR products that are usually processed with a reaction clean-up. We, therefore, explored whether transformation efficiency would be reduced in the absence of gel extraction. We subjected colonies of *E. coli* (pPICZαA) to colony PCR amplification with primers which mimic PmeI cleavage products and transformed stored, electrocompetent *P. pastoris* GS115 cells with (i) gel extracted linearised pPICZαA, (ii) gel extracted PCR product, and (iii) PCR product purified by reaction clean-up. Transformations were incubated at 30 °C and the colonies counted after 48 hours (Fig. 2). The results showed PCR products yield a higher average number of colonies than digested DNA, regardless of the purification method (Supplementary Table 2). The high variation in colony counts, e.g. the number of colonies per transformation with PmeI digested DNA ranged from 40 to 882, is a consequence of using independent batches of electrocompetent cells for each biological repeat. It indicates the expected variation in transformation efficiency, or process robustness, by incorporating fluctuations in cell concentration (final OD_600_ 0.8–1.0), competency (cell storage between one day and four weeks) and electroporation constants (4.8–5.1 ms). We carried out a three-way ANOVA on the relationship between the number of colonies and (i) the sample preparation method, (ii) the electroporation time constant and (iii) the cell batch. Results showed that while the electroporation constant (p-value = 0.00018) and cell batch (p-value = 0.00139) significantly affected the number of colonies, the sample preparation method was not statistically significant. A one-way ANOVA on the data expressed as a fold-change with respect to PmeI digest (Fig. 2), which controls for the effect of cell batch, showed no significant effect of sample preparation method (p-value = 0.312). Therefore, the use of PCR fragments did not negatively impact protocol efficacy while still reducing time and reagent costs.

One concern with amplifying PCR products for transformation directly from colonies is carryover of DNA from the colonies along with the PCR product, be it native DNA from *E. coli* or the plasmid template, particularly the antibiotic resistance cassette. To quantify this effect, PCR reactions were carried out using either the αA_PmeI_F/R primers to generate an integration cassette or the T7_F/R primers, which have no homologous target within the cells or plasmid and therefore act as a mock reaction to quantify any carryover. Spectrophotometric measurements of DNA concentration suggested some DNA was recovered from the mock reactions, an average of 19.79 ng µL^−1^ (S.D. = ± 7.44, n = 3) compared to 120.54 ng µL^−1^ (S.D. = ± 6.74, n = 3) from the real reactions (~16%). However, the ratio of absorbance at 260 nm and 230 nm was significantly lower for the mock reactions (µ = 1.07, S.D. = ± 0.26) than the real reactions (µ = 1.91, S.D. = ± 0.16), suggesting non-specific absorbance (t(4) = 4.75, p = 0.00896). DNA gel electrophoresis of the PCR reactions confirmed no visible bands in the mock reactions and while transformations of real reactions yielded colonies (µ = 85.7, S.D. = ± 38.9, n = 3), replicating the experiment with mock reactions did not yield any colonies (n = 3).

A second concern with integrating expression cassettes derived from PCR amplification is the increasing occurrence of mutations with amplicon length. Our largest insert, ceruloplasmin (3.14 kB) constitutes a 5.92 kB expression cassette. Following amplification with 25 cycles, errors from Phusion polymerase would be expected in 6.51% of products. We transformed ceruloplasmin into *P. pastoris* GS115 and amplified the integration region of four colonies. Sequencing revealed no errors, indicating that our approach generates intact expression strains quickly.

### Redesigning pPICZαA for PCR product transformation

Using PCR to produce DNA for transformation provides a further opportunity to remove extraneous vector parts that have no function in *P. pastoris*, such as those necessary for *E. coli* propagation. For integration the PCR product requires homology at the 5′ and 3′ ends, however the pUC origin is not required for growth in *P. pastoris*. By reshuffling the vector organisation we can remove the *E. coli* parts from the PCR amplicon, decreasing the chance that errors are introduced during copying or that *E. coli* vector DNA is used to “loop out” expression cassettes within the *Pichia* genome during the generation of multi-copy clones by reducing the length of homologous regions.

**Figure 3:**
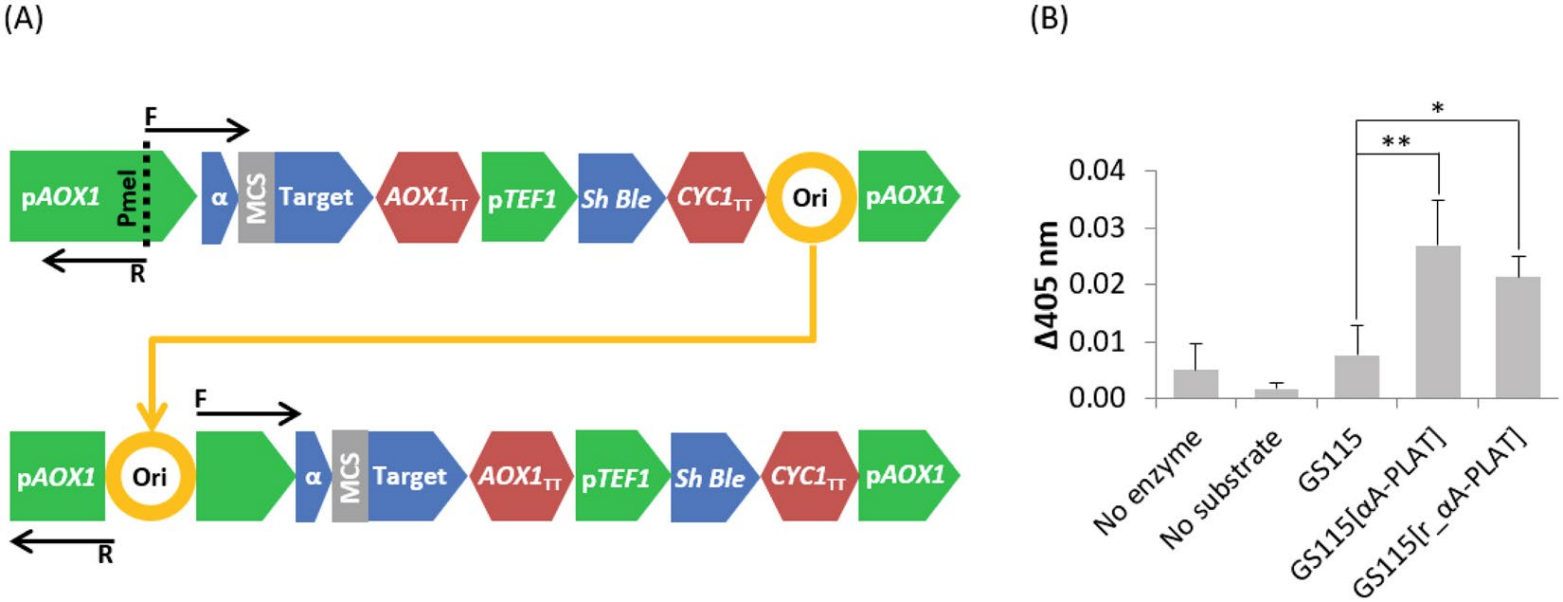
(**A**) Redesigning pPICZαA for PCR product transformation based on amplification with primers αA_PmeI_F (“F”) and αA_PmeI_R (“R”). The pUC ori region was moved from the 3′ terminus of the CYC1 transcription terminator in pPICZαA [Top] to the PmeI site of r_αA [Bottom], splitting the 5′ AOX1 promoter region. A 64 base pair reduction in size is due to removal of a 10 base pair section between CYC1 transcription terminator and the pUC ori, and a further 54 base pairs between the pUC ori and the 5′ AOX1 promoter region. Overall, the size of the PCR amplicon to produce the integration cassette is reduced by 683 bp. (**B**) TPA activity of culture supernatants from clones integrating pPICZαA-PLAT or r_αA-PLAT following 120 hours of induction. Data represent the average of six clones for each of GS115[αA-PLAT] and GS115[r_αA-PLAT]; for no enzyme, no substrate and GS115 the data are the average of three independent reactions/clones. ANOVA tests were conducted on the data; significant differences are indicated by asterisks where **p < 0.01 and *p < 0.05. Post-hoc Tukey tests revealed activity in GS115 supernatants to be significantly different from GS115[αA-PLAT] (p-value = 0.00621) and from GS115[r_αA-PLAT] (p-value = 0.0475) supernatants.

Therefore, we moved the pUC ori to the PmeI site by assembling three parts: (i) from downstream of the PmeI site in the AOX1 promoter region to the 3′ terminus of the CYC1 transcription terminator (2,495 bp), (ii) from downstream of the pUC ori to upstream of the PmeI site in the AOX1 promoter (467 bp), and (iii) the pUC ori itself (730 bp). This retained *E. coli* propagation, but reduced the PCR amplicon for integration by 683 bp (Fig. 3 (A)). Colony PCR revealed an assembly rate of ~19% (n = 16). The sequence of the reshuffled plasmid (denoted r_αA) was verified and subsequently used as a backbone to create additional TPA clones with which we could ascertain differences in PCR product transformation efficiency. Across three separate DNA preparations and cell batches, we found an average of 362 and 388 colonies per plate for pPICZαA-PLAT and r_αA-PLAT, respectively; a paired samples t-test showed no significant difference in transformation efficiency (t(2) = 4.30, p = 0.914). PCR of six colonies from each transformation confirmed 100% integration of the TPA construct. Although no difference was made to the coding sequence of the expression cassette, we wished to confirm the functionality of r_αA. We expressed TPA from six clones each of pPICZαA-PLAT and r_αA-PLAT, feeding with methanol every 24 hours for six days. TPA activity in the culture supernatant was determined with a chromogenic peptide, where cleavage is indicated by an increase in absorbance at 405 nm (Fig. 3 (B)). No significant difference was found in TPA activity in the culture supernatants from colonies transformed with the two constructs, but activity was significantly higher than supernatant from the wild-type strain GS115 (p-value = 0.0004). The reduced average activity of supernatants from GS115[r_αA-PLAT] strains may simply reflect the inherent variation between clones of *P. pastoris* observed in chromosomal integrants^10^.

### Testing the streamlined workflow with a range human therapeutic targets

**Figure 4:**
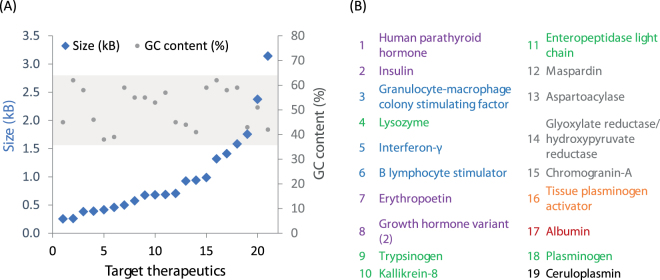
(**A**) The distribution in size and GC content across the (**B**) target proteins. The table lists the proteins in increasing size, coded by therapeutic application.

Following functional confirmation of r_αA, we wished to evaluate the efficiency of our streamlined workflow across a wide range of targets. Therefore, we selected targets with a large range of size and GC content (Fig. 4 (A)) and designed primers with exactly 50 base pairs of homology to the assembly site with melting temperatures suitable to standardised PCR reaction conditions (Supplementary Table 1). We selected targets from human cDNA which would be informative for the research and discovery aspects of the biopharmaceutical industry, either as direct therapeutics or as research targets, for instance enzyme variants/isoforms which cause genetic disorders or biomarkers of disease progression (Fig. 4 (B)). Each target was inserted directly downstream of the Kex2 cleavage sequence of the α-mating factor secretion signal in r_αA, deleting any native signal peptides which were present. In *P. pastoris*, the *S. cerevisiae* α-mating factor signal sequence is processed in two steps. Initially, Kex2 cleaves the sequence, leaving a four amino acid Glu-Ala-Glu-Ala repeat. This is subsequently processed by Ste13; however, this second cleavage is often problematic as noted in the recent extension of the Yeast ToolKit for *P. pastoris* ^17^. Therefore, by inserting targets directly following the Kex2 cleavage site and eliminating the Glu-Ala-Glu-Ala repeat we can maintain the native N-terminus of the proteins.

The parts for *E. coli* assembly were amplified in three batches due to user restrictions; however, this workflow is readily transferred to robotics for parallelisation. Initially, all backbone and insert PCRs were conducted in high fidelity (HF) enzyme buffer under identical thermocycling conditions. This amplified all vector backbones; an unsurprising result as they are identical with the exception of peripheral primer sequences. For targets, only insulin (*INS*) and granulocyte-macrophage colony stimulating factor (*CSF2*) produced no amplicons in HF buffer, but did so in GC buffer. At 62% and 58% GC content for *INS* and *CSF2*, respectively, they were among targets with the highest GC content. However, this was not predictive, as chromagranin-A, glyoxylate reductase/hydroxypyruvate reductase (*GRHPR*) and TPA had 62, 59 and 59% GC content, respectively, and still could be amplified in HF buffer.

Following each assembly, eight *E. coli* colonies were subjected to colony PCR: a minimum of one and a maximum of seven positive colonies were observed, with an average mean and median of 46.7% and 50%, respectively (Supplementary Table 3). Previous authors have anecdotally noted that the assembly efficiency decreases at the extremes of insert size^15^; however, here a multiple linear regression analysis did not show any influence of size or GC content on assembly success (F-statistic: 0.123, p-value: 0.886). One factor which could alter the rate of assembly is the presence of non-target amplicons in PCR reactions. Here multiple amplicons were only generated for *GPHPR* (2 products) and *CFS2* (3 products). We applied the same protocol for cloning with no additional purification steps. Colony PCR identified 25% and 62.5% correct colonies respectively, with bands sizes consistent with either insertion of the correct target or parental plasmid. With only two examples, there was not enough data to comment on any deleterious effect.

### Extending the characterisation to three-part assemblies

These results show that we have developed and characterised a standardised protocol for assembling single target inserts into a stock expression vector and creating *P. pastoris* strains. While this is applicable to the majority of cloning and expression experiments in *P. pastoris*, we were also interested in including higher order, multi-part assemblies in this characterisation. These are not only applicable to the bicistronic expression of traditional monoclonal antibodies, the leading and most lucrative product type in biopharmaceutical production, but they are also pertinent to the generation of ‘biobetters’ which are seeing increasing rates of approval^1^. This includes next-generation antibodies, such as antibody-fusions, conjugates and derivative fragments but also non-antibody biologics such as Albiglutide, where fusion of albumin to the glucagon-like peptide-1 agonist extends the half-life for treatment of type 2 diabetes^18^.

Monoclonal antibodies consist of two heavy chains and two lights chains, bound by disulphide bonds. As an example, we cloned the broadly neutralising anti-HIV antibody VRC01^19^. As in previous work by others^9^, we took advantage of the 2A self-processing peptide to express both chains from a single expression cassette. First discovered in 1991 in the foot-and-mouth-disease virus^20^, 2A peptides are now gaining prominence particularly for the expression of multi-enzyme pathways, as they remove the recombination risk introduced by repeated promoter and terminator sequences. Here, we separated the 5′ light (V_L_) chain from the 3′ heavy (V_H_) chain genes with the codon optimised T2A sequence from *Thosea asigna* virus, which was encoded in the primer sequences and was selected based on previous results showing a lower variance in processing efficiency^21^. The murine secretion signals previously used for expression^22^ were amplified concurrent with both chains, and the backbone amplification moved upstream of the α-mating factor secretion signal to remove it from the final construct. The three parts of r_αA (3177 bp), VL-T2A (763 bp) and T2A-VH (1497 bp) were amplified and used to transform *E. coli*; colony PCR results revealed a 25% assembly rate (n = 8) and a candidate was confirmed by sequencing.

Although antibodies have previously been expressed in *P. pastoris*, microbial hosts are generally suited to smaller single polypeptide proteins such as scFvs. As a neutralising antibody, VRC01 binds the HIV-1 surface envelope gp120 glycoprotein preventing its interaction with the CD4 receptor found on host cells^23^. This action does not require the Fc region for the subsequent recruitment of effector cells or the complement and as such could benefit from expression in a reduced format. Indeed, smaller antibody fragments are less hindered by steric interference and can reach epitopes more efficiently than the corresponding full-length parental antibody, as shown for the related anti-HIV Fab fragment X5^24^. Therefore, we wished to demonstrate the potential for this workflow in early research and discovery phases of biopharmaceutical production by generating *P. pastoris* strains expressing scFv variants from VRC01 and analysing their yield.

The variable regions of the heavy and light chains were identified through an NCBI conserved domain search^25^ and cross-checked against those identified by Wu *et al*.^19^. We designed four variants of the VRC01 scFv, each containing a codon optimised (Gly_4_Ser)_3_ linker. As the domain orientation (V_H_-V_L_ versus V_L_-V_H_) and the presence of N- or C-terminal affinity tags are well known to affect yield and activity of scFvs, we designed every combination: (i) V_H_-V_L_-tag, (ii) V_L_-V_H_-tag, (iii) tag-V_H_-V_L_ and (iv) tag-V_L_-V_H_. In the first iteration, we carried out two three-part reactions to assemble V_H_-V_L_ in r_αA (486 bp, 376 bp and 3238 bp, respectively) and V_L_-V_H_ in r_αA (426 bp, 428 bp and 3238 bp, respectively). A single murine secretion signal was amplified concurrently with the N-terminal variable domain, the (Gly_4_Ser)_3_ linker was encoded in the primer sequences and provided homology between the two domains, and the C-terminal tags from the vector were maintained by not encoding a stop codon at the end of the domain. In the second iteration, we encoded the tag sequence into the assembly primers and moved it to the N-terminus, immediately following the murine secretion signal, in a two-part assembly with each scFv constructed previously as a single part. In contrast to the other 23 assemblies conducted in this research (19 single insert assemblies and the three-part assemblies for r_αA, VRC01 and two scFvs), the *in vivo* assembly of the scFvs with N-terminal tags was very inefficient. An initial PCR screen of eight colonies from tag-V_H_-V_L_ (r_αA: 3271 bp, V_H_-V_L_: 797 bp) and tag-V_L_-V_H_ (r_αA: 3274 bp, V_L_-V_H_: 793 bp) each showed no correct assemblies. To provide a useful comparison to researchers in the field, we concurrently re-assembled tag-V_H_-V_L_ and tag-V_L_-V_H_ from the same PCR products with both *in vivo* and Gibson assembly protocols. A colony PCR screen from each of the four transformations revealed that Gibson assembly produced 100% (n = 8) and 25% (n = 8) correct assemblies for tag-V_H_-V_L_ and tag-V_L_-V_H_ respectively, while *in vivo* assembly only yielded 4.17% (n = 24) and 0% (n = 32), respectively and required much larger screens overall.

**Figure 5:**
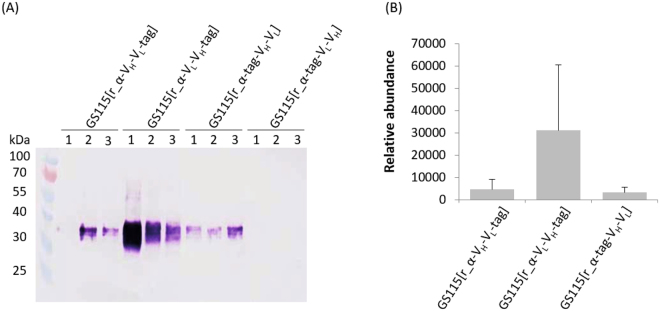
Expression of the single-chain antibody fragment derivatives of the monoclonal antibody VRC01 from *Pichia pastoris* GS115. (**A**) Western blot of culture supernatant; detection employed a primary anti-His Tag antibody and an anti-mouse secondary antibody. For each strain, three clones were cultivated in 50 mL culture tubes at 20 °C for 72 hours, feeding with 0.5% methanol every 24 hours. The expected size of the scFvs is 30 kDa; the increase in size is anticipated to be the result of N-linked glycosylation at the Asn-Leu-Thr glycosylation sequon in the light chain. (**B**) Densitometric analysis of the Western blot in (**A**) using ImageJ.

To complete the proof-of-concept, we used primers αA_PmeI_F and αA_PmeI_R to amplify the expression cassette (Fig. 3 (A)) from confirmed r_αA-VRC01 and r_αA-scFv colonies and used this to transform electrocompetent *P. pastoris* cells. Curiously, transformation of r_αA-VRC01 into GS115 produced colonies with integrants at the *AOX1* locus (as verified by colony PCR) but amplicons were ~1 kB shorter than predicted. We transformed the same DNA into stored, electrocompetent *Δku70* cells, which resolved the issue. The Ku70p binds to double-strand breaks and helps to mediate non-homologous end joining; the knockout therefore has increased homologous recombination efficiency^26^. Analysis of VRC01 expression by Western blot showed successful secretion of the separate heavy and light chains from the bicistronic T2A vector in five of six colonies tested with no detectable secretion of the unprocessed, full length peptide (Supplementary Figure 1). In one colony no heavy chain secretion was detected, although levels of the light chain were consistent with the other cultivations. Analysis of the scFv derivatives showed an increased abundance of V_L_-V_H_-tag in comparison to V_H_-V_L_-tag and tag-V_H_-V_L_; while no tag-V_L_-V_H_ was detected (Fig. 5). An ANOVA test showed that there was no statistically significant difference between the secretion levels of the different configurations (p-value = 0.109), a likely consequence of the inter-clonal variation inherent in *P. pastoris* expression, previously noted with the TPA expression results^10^. A previous study has, however, noted the poor expression of a VRC01 scFv in the V_H_-(Gly_3_Ser)_4_-V_L_ arrangement from a transient six day suspension culture of HEK 293–6E cells^27^ suggesting that the V_L_-V_H_-tag configuration is preferable.

## Discussion

Here, we have shown that the extraordinarily simple *E. coli* self-assembly method is applicable to standardised cloning of a wide range of therapeutic targets from human cDNA, demonstrating explorative product discovery. Additionally, the method can be extended to multi-part assembly, as evidenced in the reshuffling of the pPICZαA vector and the cloning of VRC01 and the scFv derivatives. At the outset we wished to characterise the efficiency of this process to give an indication as to its reliability over a wide range of targets. We found an average mean and median of 46.7% and 50% assembly success over the single insert targets. We did, however, notice an extremely low efficiency of assembly for the scFvs with N-terminal tags, even though these were designed as a single part assembly, suggesting that sequence context does play a role. To provide a comparison useful to researchers in the field, we tested the same parts with Gibson assembly, which did generate the constructs with a high frequency. As our current knowledge of the molecular pathways underlying these assembly methods is lacking, it is difficult to speculate why these particular single insert constructions failed. In this particular case, as the N-terminal tags were incorporated into the primers (63 bp), it may be that a combination of incomplete primer extension and the increased frequency of self-annealing sites in longer primers caused single-strand secondary structures that prevented *in vivo* ligation. In such a case, a Gibson assembly at 50 °C would reduce these interactions, while simultaneously filling in and ligating the seams between the parts.

We combined this simplified cloning method with PCR product transformation of stored, electrocompetent *P. pastoris*. Traditionally, transformation uses 1–5 µg linearised DNA, requiring culture time, preparation of plasmid DNA, enzymatic digestion and gel purification; here we generate DNA for transformation in a single PCR reaction from the same initial colony. In addition, the transformation procedure we used has lower DNA requirements. We routinely transformed 200 ng of DNA in our experiments, which would also be obtainable via overlap-extension PCR, e.g. to create multipart assemblies of antibody variants^9^. Superficially, this process reduces labour and reagents. More importantly, however, it limits extraneous DNA from being integrated into the expression host. A recent study revealed the deleterious effects from unwanted integration of *E. coli* DNA into *P. pastoris* during transformation and recommended PCR amplification prior to transformation to reduce these effects^28^. We have advanced on this by redesigning the vector to remove any unwanted *E. coli* DNA from the expression cassette, further limiting the risk of integration.

The streamlined approach to generating *P. pastoris* expression clones benefits from reduced labour, equipment time, enzymes, molecular biology kits; and, importantly, can be considered a platform process that requires limited troubleshooting. While the estimated cost of reagents for the streamlined process is ~30% more expensive, almost one third of this is attributable to primer synthesis which will become less limiting in the future (Supplementary Table 4). Moreover, the cost analysis does not take into account reduced labour costs and those associated with reagent expiry to which restriction cloning and ligation are more susceptible. We hope this research will complement recent efforts to bring a synthetic approach to biopharmaceutical production with *Pichia pastoris*, such as recently reported work on the restriction site-free cloning vector system for testing different affinity tags and fusion proteins^8^ and the generation of episomal expression vectors^10,29^.

## Methods

### *E. coli* assembly

Therapeutic targets, with the exception of VRC01 and the scFvs, were amplified from the MegaMan Human Transcriptome Library (Agilent, Stockport, UK). The αA backbone was amplified from pPICZαA plasmid and the r_αA backbone was amplified from r_αA plasmid. VRC01 heavy and light chains were amplified from the constructs pPICZ-VRC01-H and pJAN-VRC01-L kindly provided by Dr. Rochelle Aw (Imperial College London). The T2A sequence from the *Thosea asigna* virus (EGRGSLLTCGDVEENPGP) was codon optimised *in silico* by IDT and incorporated into primer sequences for amplifying the light and heavy chains. The murine secretion signals were amplified concurrently with the coding sequence, and the α-mating factor secretion signal deleted from the r_αA backbone. The (Gly_4_Ser)_3_ linker used to fuse the light and heavy variable domains of the scFvs was codon optimised *in silico* by IDT and incorporated into primer sequences.

All primers (Life Technologies, Paisley, UK, see Supplementary Table 1) contained exactly 50 base pairs of homology, encompassing the insertion site, with the exception of the T2A and (Gly_4_Ser)_3_ sequences where the whole part was used as the basis for homology (54 bp and 45 bp, respectively). Primer melting temperatures were calculated from the section homologous to the template with the T_m_ calculator for Phusion polymerase (Thermo Fisher Scientific, Paisley, UK). Each target was first amplified in a single 50 µL PCR reaction mix, containing 1X Phusion HF buffer, 10 mM dNTPs, 10 µM forward primer, 10 µM reverse primer, 2% DMSO, 1U Phusion DNA polymerase and either 50 ng plasmid backbone or 0.5 µL human cDNA. Two of the targets (Insulin and Granulocyte-macrophage colony stimulating factor) did not amplify in HF buffer, but were successfully amplified in GC buffer.

With the exception of preliminary testing, all target therapeutics were cloned into the plasmid r_αA. The plasmid is identical in sequence composition to the plasmid pPICZαA (Invitrogen, Life Technologies, Paisley, UK), however the ColE1 origin of replication has been moved to the PmeI cut site, splitting the region of 5′AOX1 promoter homology. For the sequences amplified from human cDNA, each target was inserted into the same site in r_αA, immediately following the arginine residue at the 5′ side of the Kex2 cleavage site, downstream of the *S. cerevisiae* α-mating factor secretion signal. The 3′ terminus of the insert finishes two amino acids prior to the vector Myc tags, removing 73 base pairs of the multiple cloning site. Expression of the antibody and scFvs utilised the murine secretion signal, inserting the sequences immediately downstream of the *AOX1* promoter region and replacing the α-mating factor secretion signal.

All PCR products for assembly were digested with DpnI (NEB, Hitchin, UK), with 1 µL enzyme added directly to the PCR reaction and incubated at 37 °C for one hour. The products were cleaned with Zymo DNA Clean & Concentrator™-5 columns (Cambridge Bioscience, Cambridge, UK) and eluted in 10 µL Milli-Q purified water. The DNA concentration and purity were analysed by NanoDrop^TM^ (ThermoFisher Scientific, Wilmington, DE, USA) and 0.8% TAE-agarose gels.

The PCR parts were assembled in NEB5α Competent *E. coli* (High Efficiency) cells (NEB, Hitchin, UK). Each 50 µL vial was divided equally between two to four, pre-chilled 2 mL polypropylene tubes. DNA parts were mixed with the cells at a molar concentration of 5:1 (insert:backbone) and transformed according the manufacturers’ recommendations. Cells were selected on low salt LB agar plates containing 100 µg mL^−1^ Zeocin (Life Technologies, Paisley, UK). For Gibson assembly, parts were added to the isothermal mastermix at the same molar concentration^13^ and incubated at 50 °C for an hour; 2 µL was used in subsequent transformations.

Colony PCR was used to confirm target insertion, using REDTaq® Readymix (Sigma, Dorset, UK) and primers AOX1_col_F and AOX1_col_R which bind in the 5′ AOX1 promoter region and the 3′ AOX1 transcription termination region, respectively. DNA was extracted from positive colonies with a miniprep kit (Qiagen, Manchester, UK) and sequenced (Eurofins Genomics, Ebersberg, Germany).

### *Pichia pastoris* transformation

PCR products for transformation were amplified from a single *E. coli* colony in a 50 µL PCR Phusion reaction, as previously described, with primers αA_PmeI_F and αA_PmeI_R. The PCR reaction was either cleaned using Zymo DNA Clean & Concentrator™-5 columns, or gel extracted from a 0.8% TAE-agarose gel using the Zymoclean™ Gel DNA Recovery Kit (Cambridge Bioscience, Cambridge, UK), and eluted in 10 µL Milli-Q purified water.

*P. pastoris* (GS115 and Δku70 (CBS 12694, CBS-KNAW, Fungal Biodiversity Centre, Utretcht, The Netherlands) cells were transformed with 200 ng DNA according to the protocol of Lin-Cereghino *et al*.^7^ with the following alterations. The 10 mM bicine-NaOH, pH 8.3 and 3% (v/v) ethylene glycol in the BEDS solution was replaced with tricine-NaOH and glycerol, at the same concentration and pH, respectively. Cells from a 50 mL culture were resuspended in a final volume of 500 µL BEDS solution (0.01 volumes). Transformations were recovered in Yeast Extract Peptone Dextrose medium with Sorbitol (YPDS) at 30 °C, 225 rpm for exactly one hour and plated onto YPD agar plates containing 100 µg mL^−1^ Zeocin.

Colony PCR with primers AOX1_col_F and AOX1_col_R was used to confirm clones which had integrated the heterologous expression cassette. Each colony was resuspended in 20 μL 20 mM NaOH and incubated for 15 minutes at 100 °C. From the supernatant, 2 µL was used as a template for a 20 µL Phusion PCR as previously described.

### Small scale expression and analysis by Western blot

For small scale expression, colonies from a fresh transformation plate were inoculated into 5 mL YPD containing 100 µg mL^-1^ zeocin and incubated at 30 °C, 250rpm overnight. The OD_600_ was measured and each culture standardised to an OD_600_ of 1.0 in 4 mL BMMY (50 mL culture tube) or 3 mL BMMY (24 well plate). For tissue plasminogen activator expression, the induced cultures were incubated at 30 °C 250 rpm for six days, feeding with a final concentration of 0.5% methanol every 24 hours. At 72, 96 and 120 hours supernatant samples were taken for TPA expression analysis. For VRC01 and scFv expression, induced cultures were incubated at 20 °C 250 rpm for three days, feeding with a final concentration of 0.5% methanol every 24 hours. Supernatant samples were analysed by 12% Tris-HCl SDS-PAGE and subsequently transferred to an Immobilon-FL polyvinylidene fluoride membrane (Millipore, Watford, UK) using a Novex® Semi-Dry Blotter (Thermo Fisher Scientific, Paisley, UK). VRC01 antibody was detected with an AP-conjugated Rabbit Anti-Human IgG H&L (Abcam, Cambridge, UK) at a concentration of 1:5000 and developed with the alkaline phosphate substrate BCIP/NBT kit (Thermo Fisher Scientific). All other proteins were detected with a purified anti-His Tag Antibody [Clone: J099B12] (BioLegend UK, London, UK) at a concentration of 1:1000 an developed with the anti-mouse WesternBreeze® Chromogenic Kit (Thermo Fisher Scientific, Paisley, UK).

### Tissue Plasminogen Activator (TPA) activity assay

The activity of TPA in *P. pastoris* culture supernatants was assessed with a chromogenic substrate (Sigma, Dorset, UK). Reactions consisting of 219 µL 50 mM Tris-HCl pH 8, 25 µL 2 mM substrate (solubilised in dH_2_O) and 6.25 µL culture supernatant were assembled in a 96 well plate and the absorbance measured at 405 nm. The plate was sealed and incubated for 16 hours at 37 °C after which the final absorbance reading was measured.

### Data availability

All data generated or analysed during this study are included in this published article (and its Supplementary Information files) and can be used without restriction.

## Electronic supplementary material

Supplementary Information

## References

[1] Walsh G (2014). Biopharmaceutical benchmarks. Nat. Biotech..

[2] Parsaie Nasab F, Aebi M, Bernhard G, Frey ADA (2013). Combined System for Engineering Glycosylation Efficiency and Glycan Structure in *Saccharomyces cerevisiae*. Appl. Environ. Microbiol..

[3] Kurtzman CP (2005). Description of *Komagataella phaffii* sp. nov. and the transfer of *Pichia pseudopastoris* to the methylotrophic yeast genus. Komagataella. Int. J. Syst. Evol. Microbiol..

[4] Zhang N (2011). Glycoengineered *Pichia* produced anti-HER2 is comparable to trastuzumab in preclinical study. mAbs.

[5] Vogl T (2016). A Toolbox of Diverse Promoters Related to Methanol Utilization: Functionally Verified Parts for Heterologous Pathway Expression in *Pichia pastoris*. ACS Synth. Biol..

[6] Weninger, A., Glieder, A. & Vogl, T. A toolbox of endogenous and heterologous nuclear localization sequences for the methylotrophic yeast *Pichia pastoris*. *FEMS Yeast Res*. **15**, 10.1093/femsyr/fov082 (2015). 10.1093/femsyr/fov082 PMC4629791 26347503

[7] Lin-Cereghino J (2005). Condensed protocol for competent cell preparation and transformation of the methylotrophic yeast *Pichia pastoris*. BioTechniques.

[8] Vogl T, Ahmad M, Krainer FW, Schwab H, Glieder A (2015). Restriction site free cloning (RSFC) plasmid family for seamless, sequence independent cloning in *Pichia pastoris*. Microb Cell Fact..

[9] Shah KA (2015). Automated pipeline for rapid production and screening of HIV-specific monoclonal antibodies using *Pichia pastoris*. Biotechnol. Bioeng..

[10] Camattari A (2016). Characterization of a panARS-based episomal vector in the methylotrophic yeast *Pichia pastoris* for recombinant protein production and synthetic biology applications. Microb Cell Fact..

[11] Bubeck P, Winkler M, Bautsch W (1993). Rapid cloning by homologous recombination *in vivo*. Nucleic Acids Res..

[12] Oliner JD, Kinzler KW, Vogelstein B (1993). *In vivo* cloning of PCR products in *E. coli*. Nucleic Acids Res..

[13] Gibson DG (2009). Enzymatic assembly of DNA molecules up to several hundred kilobases. Nat. Methods.

[14] Beyer HM (2015). AQUA Cloning: A Versatile and Simple Enzyme-Free Cloning Approach. PLoS One.

[15] Kostylev M, Otwell AE, Richardson RE, Suzuki Y (2015). Cloning Should Be Simple: *Escherichia coli* DH5α-Mediated Assembly of Multiple DNA Fragments with Short End Homologies. PLoS One.

[16] Invitrogen. *Pichia* Expression Kit: For Expression of Recombinant Proteins in *Pichia pastoris* (2010).

[17] Obst, U., Lu, T. K. & Sieber, V. A modular toolkit for generating *Pichia pastoris* secretion libraries. *ACS Synth. Biol*., 10.1021/acssynbio.6b00337 (2017). 10.1021/acssynbio.6b00337 28252957

[18] Trujillo JM, Nuffer W (2014). Albiglutide: a new GLP-1 receptor agonist for the treatment of type 2 diabetes. Ann. Pharmacother..

[19] Wu X (2010). Rational Design of Envelope Identifies Broadly Neutralizing Human Monoclonal Antibodies to HIV-1. Science.

[20] Ryan MD, King AM, Thomas GP (1991). Cleavage of foot-and-mouth disease virus polyprotein is mediated by residues located within a 19 amino acid sequence. J. Gen. Virol..

[21] Geier M, Fauland P, Vogl T, Glieder A (2015). Compact multi-enzyme pathways in *P. pastoris*. Chem. Commun. (Camb.).

[22] Aw R, McKay PF, Shattock RJ, Polizzi KM (2017). Expressing anti-HIV VRC01 antibody using the murine IgG1 secretion signal in *Pichia pastoris*. AMB Express.

[23] Li Y (2011). Mechanism of Neutralization by the Broadly Neutralizing HIV-1 Monoclonal Antibody VRC01. J. Virol..

[24] Labrijn AF (2003). Access of Antibody Molecules to the Conserved Coreceptor Binding Site on Glycoprotein gp120 Is Sterically Restricted on Primary Human Immunodeficiency Virus Type 1. J. Virol..

[25] Marchler-Bauer A (2017). CDD/SPARCLE: functional classification of proteins via subfamily domain architectures. Nucleic Acids Res..

[26] Näätsaari L (2012). Deletion of the *Pichia pastoris* KU70 Homologue Facilitates Platform Strain Generation for Gene Expression and Synthetic Biology. PLoS One.

[27] West AP, Galimidi RP, Gnanapragasam PNP, Bjorkman PJ (2012). Single-Chain Fv-Based Anti-HIV Proteins: Potential and Limitations. J. Virol..

[28] Schwarzhans JP (2016). Non-canonical integration events in *Pichia pastoris* encountered during standard transformation analysed with genome sequencing. Sci. Rep..

[29] Schwarzhans JP (2017). A Mitochondrial Autonomously Replicating Sequence from *Pichia pastoris* for Uniform High Level Recombinant Protein Production. Front. Microbiol..

[30] Parrish JR (2004). High-Throughput Cloning of *Campylobacter jejuni* ORFs by *In Vivo* Recombination in *Escherichia coli*. J. Proteome Res..

[31] Li C (2011). Fast Cloning: a highly simplified, purification-free, sequence- and ligation-independent PCR cloning method. BMC Biotechnol..

[32] Jacobus AP, Gross J (2015). Optimal Cloning of PCR Fragments by Homologous Recombination in *Escherichia coli*. PLoS One.

[33] Garcia-Nafria J, Watson JF, Greger IH (2016). IVA cloning: A single-tube universal cloning system exploiting bacterial *In Vivo* Assembly. Sci. Rep..

[34] Liu CJ (2017). OEPRCloning: An Efficient and Seamless Cloning Strategy for Large- and Multi-Fragments. Sci. Rep..

