# Supplementary material for: A streamlined cloning workflow minimising the time-to-strain pipeline for Pichia pastoris
Source: Sci Rep. 2017 Nov 17;7:15817. doi: 10.1038/s41598-017-16172-0 (PMC5693959; doi:10.1038/s41598-017-16172-0)
Supplement: Supplementary file 1 — Supplementary Information [file 41598_2017_16172_MOESM1_ESM.pdf]

SUPPLEMENTARY INFORMATION

A streamlined workflow minimising time-to-strain pipelines in *Pichia pastoris*

Kate E. Royle[1,2] & Karen Polizzi[1,2]*

1. Department of Life Sciences, Imperial College London
2. Centre for Synthetic Biology and Innovation, Imperial College London

Table S1: Primers used in this study. Names denote the target amplified with any homology regions in square brackets. 'F' and 'R' denote forward and reverse primers, while 'col' indicates primers for colony PCR and/or sequencing.

Primer Name	Primer Sequence
Testing the assembly efficiency with different locations of homology	
αA_[PLAT]_F	[GATTCGTGACAACATGCGACCG]TTTCTAGAACAAAAACTCATCTCAGAAG
αA_[PLAT]_R	[TTTCATCTCTGCAGATCACTTGGTAAGA]TCTTTTCTCGAGAGATACCCCT
PLAT_[αA]_F	[AAGGGGTATCTCTCGAGAAAAGA]TCTTACCAAGTGATCTGCAGAGATGAA
PLAT_[αA]_R	[TCCTCTTCTGAGATGAGTTTTTGTTCTAGAAA]CGGTCGCATGTTGTCACG
αA_[]_F	TTTCTAGAACAAAAACTCATCTCAGAAG
αA_[]_R	GAATTCAGCTTCAGCCTCTCTT
PLAT_[αA]_ F2	[CTATTGCCAGCATTGCTGCTAAAGAAGAAGGGGTATCTCTCGAGAAAAGA]TCTTACCAAG TGATCTGCAGAGATGAA
PLAT_[αA]_ R2	[TCGACGGCGCTATTCAGATCCTCTTCTGAGATGAGTTTTTGTTCTAGAAA]CGGTCGCATGT TGTCACG
αA_[TNFSF13B]_F	[TTTTGGTGCATTGAAACTGCTG]TTTCTAGAACAAAAACTCATCTCAGAAG
αA_[TNFSF13B]_R	[TGACTGTTTCTTCTGGACCCTGAACGGC]TCTTTTCTCGAGAGATACCCCT
TNFSF13B_[αA]_F	[TAAAGAAGAAGGGGTATCTCTCGAGAAAAGA]GCCGTTCAGGGTCCAGAAG
TNFSF13B_[αA]_R	[CTTCTGAGATGAGTTTTTGTTCTAGAAA]CAGCAGTTTCAATGCACCAAAA
TNFSF13B_[αA]_F2	[CTATTGCCAGCATTGCTGCTAAAGAAGAAGGGGTATCTCTCGAGAAAAGA]GCCGTTCAGG GTCCAGAAG
TNFSF13B_[αA]_R2	[TCGACGGCGCTATTCAGATCCTCTTCTGAGATGAGTTTTTGTTCTAGAAA]CAGCAGTTTCAA TGCACCAAAA
AOX1_col_F	GACTGGTTCCAATTGACAAGC
AOX1_col_R	GCAAATGGCATTCTGACATCC
Amplifying the vectors for PCR product-based transformation of *P. pastoris*	
αA_PmeI_F	AAACGCTGTCTTGGAACCTA
αA_PmeI_R	AAACTGTCAGTTTTGGGCCAT
T7_F	TAATACGACTCACTATAGGG
T7_R	GCTAGTTATTGCTCAGCGG
Assembling r_αA	
3'PmeI_[pUC]_F	[AAGATCCTTTGATCTTTTCTACGGG]AAACGCTGTCTTGGAACCTAATATG
3'PmeI_[5'PmeI]_R	[CAACCTTTCGTCTTTGGATGTTAGATCT]AGCTTGCAAATTAAAGCCTTCG
5'PmeI_[3'PmeI]_F	[GCTCGAAGGCTTTAATTTGCAAGCT]AGATCTAACATCCAAAGACGAAAGG
5'PmeI_[pUC]_R	[CTGGCCTTTTGCTGGCCTTTTGCTCACAT]AAACTGTCAGTTTTGGGCCAT
pUC_[5'PmeI]_F	[TGTTCCCCAAATGGCCCAAAACTGACAGTTT]ATGTGAGCAAAAGGCCAGC
pUC_[3'PmeI]_R	[CATATTAGGTTCCAAGACAGCGTTT]CCCGTAGAAAAGATCAAAGGATCTT
r_αA_col_F	AATTTGCAAGCTAGATCTAACATC
r_αA_col_R	GACAGCGTTTCCCGTAGAAA

Cloning the therapeutic targets	
αA_[PTH]_F	[ATTAACTAAAGCTAAATCCCAG]TTTCTAGAACAAAAACTCATCTCAGAAG
αA_[PTH]_R	[TATGCATAAGCTGTATTTCACTCACAGA]TCTTTTCTCGAGAGATACCCCT
PTH_[αA]_F	[AAGGGGTATCTCTCGAGAAAAGA]TCTGTGAGTGAAATACAGCTTATGCAT
PTH_[αA]_R	[GAGTTTTTGTTCTAGAAA]CTGGGATTTAGCTTTAGTTAATACATTCACAT
αA_[INS]_F	[CCAGCTGGAGAACTACTGCAAC]TTTCTAGAACAAAAACTCATCTCAGAAG
αA_[INS]_R	[GTGAGCCGCACAGGTGTTGGTTCACAAA]TCTTTTCTCGAGAGATACCCCT
INS_[αA]_F	[AAGAAGAAGGGGTATCTCTCGAGAAAAGA]TTTGTGAACCAACACCTGTGC
INS_[αA]_R	[CTGAGATGAGTTTTTGTTCTAGAAA]GTTGCAGTAGTTCTCCAGCTGGTAG
αA_[CSF2]_F	[CTGCTGGGAGCCAGTCCAGGAG]TTTCTAGAACAAAAACTCATCTCAGAAG
αA_[CSF2]_R	[TGCTGGGGCTGGGCGAGCGGGCGGGTGC]TCTTTTCTCGAGAGATACCCCT
CSF2_[αA]_F	[GCTGCTAAAGAAGAAGGGGTATCTCTCGAGAAAAGA]GCACCCGCCCGCTC
CSF2_[αA]_R	[CCTCTTCTGAGATGAGTTTTTGTTCTAGAAA]CTCCTGGACTGGCTCCCAG
αA_[LYZ]_F	[GTATGTTCAAGGTTGTGGAGTG]TTTCTAGAACAAAAACTCATCTCAGAAG
αA_[LYZ]_R	[TGGCCAACTCACACCTTTCAAAGACCTT]TCTTTTCTCGAGAGATACCCCT
LYZ_[αA]_F	[AAGAAGGGGTATCTCTCGAGAAAAGA]AAGGTCTTTGAAAGGTGTGAGTTG
LYZ_[αA]_R	[TCTGAGATGAGTTTTTGTTCTAGAAA]CACTCCACAACCTTGAACATACTG
αA_[IFNG]_F	[TCAGATGCTGTTTCGAGGTCGA]TTTCTAGAACAAAAACTCATCTCAGAAG
αA_[IFNG]_R	[TTTCTGCTTCTTTTACATATGGGTCCTG]TCTTTTCTCGAGAGATACCCCT
IFNG_[αA]_F	[AGAAGGGGTATCTCTCGAGAAAAGA]CAGGACCCATATGTAAAAGAAGCAG
IFNG_[αA]_R	[CTCTTCTGAGATGAGTTTTTGTTCTAGAAA]TCGACCTCGAAACAGCATCT
αA_[EPO]_F	[GGCCTGCAGGACAGGGGACAGA]TTTCTAGAACAAAAACTCATCTCAGAAG
αA_[EPO]_R	[GGCTGTCACAGATGAGGCGTGGTGGGGC]TCTTTTCTCGAGAGATACCCCT
EPO_[αA]_F	[CTGCTAAAGAAGAAGGGGTATCTCTCGAGAAAAGA]GCCCCACCACGCCTC
EPO_[αA]_R	[CCTCTTCTGAGATGAGTTTTTGTTCTAGAAA]TCTGTCCCCTGTCCTGCAG
αA_[GH-V1]_F	[TGTGGAGGGCAGCTGTGGCTTC]TTTCTAGAACAAAAACTCATCTCAGAAG
αA_[GH-V1]_R	[AAAGCCTGGATAAGGGAATGGTTGGGAA]TCTTTTCTCGAGAGATACCCCT
GH-V1_[αA]_F	[AAGAAGAAGGGGTATCTCTCGAGAAAAGA]TTCCCAACCATTCCCTTATCC
GH-V1_[αA]_R	[TCCTCTTCTGAGATGAGTTTTTGTTCTAGAAA]GAAGCCACAGCTGCCCTC
αA_[PRSS1]_F	[GAACACCATAGCTGCCAATAGC]TTTCTAGAACAAAAACTCATCTCAGAAG
αA_[PRSS1]_R	[TCTCCTCACAGTTGTAGCCCCCAACGAT]TCTTTTCTCGAGAGATACCCCT
PRSS1_[αA]_F	[AAGAAGAAGGGGTATCTCTCGAGAAAAGA]ATCGTTGGGGGCTACAACTGT
PRSS1_[αA]_R	[GAGATGAGTTTTTGTTCTAGAAA]GCTATTGGCAGCTATGGTGTTCTTAAT
αA_[KLK8]_F	[GAAGATCATAGGCAGCAAGGGC]TTTCTAGAACAAAAACTCATCTCAGAAG
αA_[KLK8]_R	[GGGGTTGGCACTCATGACCCCCCAGCAC]TCTTTTCTCGAGAGATACCCCT
KLK8_[αA]_F	[TAAAGAAGAAGGGGTATCTCTCGAGAAAAGA]GTGCTGGGGGGTCATGAGT
KLK8_[αA]_R	[CTTCTGAGATGAGTTTTTGTTCTAGAAA]GCCCTTGCTGCCTATGATCTTC
αA_[ENT]_F	[ATGGATACAAAGTTTTCTACAT]TTTCTAGAACAAAAACTCATCTCAGAAG
αA_[ENT]_R	[CTTCTTTGGCATTACTTCCTCCAACAAT]TCTTTTCTCGAGAGATACCCCT
ENT_[αA]_F	[AAGAAGGGGTATCTCTCGAGAAAAGA]ATTGTTGGAGGAAGTAATGCCAAA
ENT_[αA]_R	[ATGAGTTTTTGTTCTAGAAA]ATGTAGAAAACTTTGTATCCATTCGGTAAA
αA_[MAS]_F	[TGGCATCAGCCAGGAGGAGCAG]TTTCTAGAACAAAAACTCATCTCAGAAG
αA_[MAS]_R	[AATCAGGAGAGACTTTAATCTCTCCCAT]TCTTTTCTCGAGAGATACCCCT
MAS_[αA]_F	[GGGTATCTCTCGAGAAAAGA]ATGGGAGAGATTAAAGTCTCTCCTGATTAT
MAS_[αA]_R	[CCTCTTCTGAGATGAGTTTTTGTTCTAGAAA]CTGCTCCTCCTGGCTGATG
αA_[ASPA]_F	[AAGTATTCGCTGCTGTTTACAT]TTTCTAGAACAAAAACTCATCTCAGAAG
αA_[ASPA]_R	[GTTCTTCAGCAATGTGACAAGAAGTCAT]TCTTTTCTCGAGAGATACCCCT

ASPA_[αA]_F	[AGAAGGGGTATCTCTCGAGAAAAGA]ATGACTTCTTGTCACATTGCTGAAG
ASPA_[αA]_R	[TGAGATGAGTTTTTGTTCTAGAAA]ATGTAAACAGCAGCGAATACTTTTTG
αA_[GRHPR]_F	[GATGCCTAGTGAACTCAAGCTG]TTTCTAGAACAAAAACTCATCTCAGAAG
αA_[GRHPR]_R	[ACACCTTCATGAGTCGCACCGGTCTCAT]TCTTTTCTCGAGAGATACCCCT
GRHPR_[αA]_F	[AAAGAAGAAGGGGTATCTCTCGAGAAAAGA]ATGAGACCGGTGCGACTCAT
GRHPR_[αA]_R	[TTCTGAGATGAGTTTTTGTTCTAGAAA]CAGCTTGAGTTCACTAGGCATCG
αA_[CHGA]_F	[GCTGCAGGCACTACGGCGGGGC]TTTCTAGAACAAAAACTCATCTCAGAAG
αA_[CHGA]_R	[CTTTATTCATAGGGCTGTTCACAGGGAG]TCTTTTCTCGAGAGATACCCCT
CHGA_[αA]_F	[AGAAGAAGGGGTATCTCTCGAGAAAAGA]CTCCCTGTGAACAGCCCTATGA
CHGA_[αA]_R	[AGATCCTCTTCTGAGATGAGTTTTTGTTCTAGAAA]GCCCCGCCGTAGTGC
αA_[ALB]_F	[AAGTCGAGCTGCCTTAGGCTTA]TTTCTAGAACAAAAACTCATCTCAGAAG
αA_[ALB]_R	[GATGAGCAACCTCACTCTTGTGTGCATC]TCTTTTCTCGAGAGATACCCCT
ALB_[αA]_F	[AGAAGAAGGGGTATCTCTCGAGAAAAGA]GATGCACACAAGAGTGAGGTTG
ALB_[αA]_R	[CTTCTGAGATGAGTTTTTGTTCTAGAAA]TAAGCCTAAGGCAGCTCGACTT
αA_[PLG]_F	[TGAGGGAGTGATGAGAAATAAT]TTTCTAGAACAAAAACTCATCTCAGAAG
αA_[PLG]_R	[GGGTATTCACATAGTCATCCAGAGGCTC]TCTTTTCTCGAGAGATACCCCT
PLG_[αA]_F	[GAAGGGGTATCTCTCGAGAAAAGA]GAGCCTCTGGATGACTATGTGAATAC
PLG_[αA]_R	[TGAGATGAGTTTTTGTTCTAGAAA]ATTATTTCTCATCACTCCCTCAATCC
αA_[CP]_F	[AAATGAAGACACCAAATCTGGC]TTTCTAGAACAAAAACTCATCTCAGAAG
αA_[CP]_R	[TAATTCCAATGTAATAATGCTTTTCTTT]TCTTTTCTCGAGAGATACCCCT
CP_[αA]_F	[GTATCTCTCGAGAAAAGA]AAAGAAAAGCATTATTACATTGGAATTATTGA
CP_[αA]_R	[TTCTGAGATGAGTTTTTGTTCTAGAAA]GCCAGATTTGGTGTCTTCATTTT
Assembling r_αA-VRC01 and scFvs	
αA_[VH]_F	[CTCTCCCTGTCTCCGGGTAAATGA]GTTTGTAGCCTTAGACATGACTGTTC
αA_[VL]_R	[AAGGATGATACATGACCATCCCAT]CGTTTCGAATAATTAGTTGTTTTTTG
VL_[αA]_F	[ATCAAAAAACAACTAATTATTCGAAACG]ATGGGATGGTCATGTATCATCC
VL_[T2A]_R	[AGGACCAGGGTTTTCCTCTACATCGCCACACGTCAGCAAGGAGCCTCTACCTTC]ACACTCT CCCCTGTTGAAGCT
VH_[T2A]_F	[GAAGGTAGAGGCTCCTTGCTGACGTGTGGCGATGTAGAGGAAAACCCTGGTCCT]ATGGG ATGGTCATGTATCATCCTT
VH_[αA]_R	[TGAGGAACAGTCATGTCTAAGGCTACAAAC]TCATTTACCCGGAGACAGGG
αA_[VHVL_T]_F	[GGGGACCAAGGTGGAGATC]TTTCTAGAACAAAAACTCATCTCAGAAGAGG
αA_[VHVL_T]_R	[GATACATGACCATCCCAT]CGTTTCGAATAATTAGTTGTTTTTTGATCTTC
VL_[VHVL_T]_F	[GGAGGAGGAGGAAGTGGAGGTGGTGGATCCGGAGGCGGAGGTAGT]GAAATTGTGTTGA CACAGTCTCCAG
VL_[VHVL_T]_R	[CCTCTTCTGAGATGAGTTTTTGTTCTAGAAA]GATCTCCACCTTGGTCCCC
VH_[VHVL_T]_F	[AAAAACAACTAATTATTCGAAACG]ATGGGATGGTCATGTATCATCCTTTT
VH_[VHVL_T]_R	[ACTACCTCCGCCTCCGGATCCACCACCTCCACTTCCTCCTCCTCC]GGAGACGATGACCGGG GT
αA_[VLVH_T]_F	[CACCCCGGTCATCGTCTCC]TTTCTAGAACAAAAACTCATCTCAGAAGAGG
αA_[VLVH_T]_R	[GATACATGACCATCCCAT]CGTTTCGAATAATTAGTTGTTTTTTGATCTTC
VH_[VLVH_T]_F	[GGAGGAGGAGGAAGTGGAGGTGGTGGATCCGGAGGCGGAGGTAGT]CTGGTGCAGTCTG GGGGTC
VH_[VLVH_T]_R	[TCCTCTTCTGAGATGAGTTTTTGTTCTAGAAA]GGAGACGATGACCGGGGT
VL_[VLVH_T]_F	[AAAAACAACTAATTATTCGAAACG]ATGGGATGGTCATGTATCATCCTTTT
VL_[VLVH_T]_R	[ACTACCTCCGCCTCCGGATCCACCACCTCCACTTCCTCCTCCTCC]GATCTCCACCTTGGTCCC CT
αA_[T_VHVL]_F	[CAAGGTGGAGATCTTTCTATGA]GTTTGTAGCCTTAGACATGACTGTTCCT

αA_[T_VHVL]_R	[ATGATGATGATGATGATGGTCGACGGCGCTATTCAGATCCTCTTCTGAGATGAGTTTTTGTTC]GGAATGTACACCGGTTGCAGTT
VH_[T_VHVL]_F	[GAACAAAAACTCATCTCAGAAGAGGATCTGAATAGCGCCGTCGACCATCATCATCATCATCAT]CAGGTGCAGCTGGTGCA
VL_[T_VHVL]_R	[CAGTCATGTCTAAGGCTACAAACTCA]TAGAAAGATCTCCACCTTGGTCCC
αA_[T_VLVH]_F	[CCCGGTCATCGTCTCCTTTCTATGA]GTTTGTAGCCTTAGACATGACTGTTCCT
αA_[T_VLVH]_R	[ATGATGATGATGATGATGGTCGACGGCGCTATTCAGATCCTCTTCTGAGATGAGTTTTTGTTC]TGAATGTACACCGGTTGCAGTT
VL_[T_VLVH]_F	[GAACAAAAACTCATCTCAGAAGAGGATCTGAATAGCGCCGTCGACCATCATCATCATCATCAT]GAAATTGTGTTGACACAGTCTCCAGG
VH_[T_VLVH]_R	[AACAGTCATGTCTAAGGCTACAAACTCA]TAGAAAGGAGACGATGACCGGG

Table S2: Colony count data for each of the *Pichia pastoris* transformations carried out during the development of the PCR transformation method. Each sample preparation method (PmeI digest with gel extraction, PCR with reaction clean-up (C&C) and PCR with gel extraction (GE)) was tested across four different cell batches (A to D).

Sample preparation method	Repeat	Electroporation time constant (ms)	Cell batch	Number of colonies	Fold change, relative to PmeI	Average	SD
PmeI digest	1	4.8	A	66	1.00		
	2	4.8	B	40	1.00		
	3	4.8	C	146	1.00		
	4	5.1	D	882	1.00	1.00	0.00
PCR (C&C)	1	4.9	A	121	1.83		
	2	4.9	B	119	2.98		
	3	4.8	C	323	2.21		
	4	4.8	D	645	0.73	1.94	0.93
PCR (GE)	1	4.8	A	125	1.89		
	2	4.9	B	152	3.80		
	3	4.8	C	231	1.58		
	4	4.9	D	491	0.56	1.96	1.35

Table S3: Human therapeutic targets used to test single insert assemblies into r_αA with *E. coli* self-assembly. Targets are listed in increasing size, with their associated UniProt numbers and GC content. The results from colony PCR for each cloning experiment are presented as percentage correct (n = 8), based on PCR amplicons of the expected size in comparison to the negative control.

Target	UniProt	Size (bp)	GC content (%)	% Correct
Human parathyroid hormone	P01270	255	45	87.5
Insulin	P01308	261	62	75.0
Granulocyte-macrophage colony stimulating factor	P04141	384	58	62.5
Lysozyme	P61626	393	46	37.5
Interferon-γ	P01579	417	38	25.0
B lymphocyte stimulator	Q9Y275	459	39	62.5
Erythropoetin	P01588	501	59	12.5
Growth hormone variant (2)	P01241	576	55	50.0
Trypsinogen	P07477	675	55	62.5
Kallikrein-8	Q60259	687	57	12.5
Enteropeptidase (catalytic light chain)	P98073	708	45	50.0
Maspardin	Q9NZD8	927	44	50.0
Aspartoacylase	P45381	942	41	37.5
Glyoxylate reductase/hydroxypyruvate reductase	Q9UBQ7	987	59	25.0
Chromogranin-A	P10645	1320	62	50.0
Tissue plasminogen activator	P00750	1584	59	25.0
Albumin	P02768	1758	43	50.0
Plasminogen	P00747	2376	51	75.0
Ceruloplasmin	P00450	3141	42	37.5
			Mean	46.7
			Median	50.0

Table S4: Cost analysis for the streamlined and traditional workflows for generating expression strains of *Pichia pastoris*

**Streamlined**		**Traditional**	
**Step**	**Cost**	**Step**	**Cost**
Forward and reverse primers, vector & insert, 50 bp each [1]	£52.00	Forward and reverse primers, insert, 25 bp each [1]	£13.00
PCR, vector & insert [2]	£1.58	PCR, insert [2]	£0.79
DpnI, vector & insert [3]	£0.11	Isolation of vector DNA [8]	£1.37
PCR reaction clean-up, vector & insert [4]	£2.78	Restriction digest with two enzymes, vector & insert [9]	£0.02
DNA quantification with NanoDrop	£0.00	1% Agarose gel [10, 11, 12]	£4.88
E. coli transformation [5]	£2.08	Gel purification [13]	£2.88
Colony PCR with high fidelity polymerase, 8 20 µL reactions [2]	£2.52	DNA quantification with NanoDrop	£0.00
PCR reaction clean-up, vector & insert [4]	£1.39	Ligation reaction [14]	£0.56
Sequencing reactions, forward & reverse [6]	£17.62	*E. coli* transformation [5]	£2.08
Pichia transformation [7]	£4.56	Colony PCR with low fidelity polymerase, 8 reactions 20 µL [15]	£5.94
		Isolation of vector DNA [8]	£1.37
		Sequencing reactions, forward & reverse [6]	£17.62
		Restriction digest to linearise plasmid [16]	£0.12
		1% Agarose gel [10, 11, 12]	£4.88
		Gel purification [13]	£1.44
		Pichia transformation [7]	£4.56
**TOTAL**	**£84.64**	**TOTAL**	**£61.51**

Commercial list prices, valid on 12th June 2017; conversions based on €1 = £0.89. No general molecular biology reagents and consumables have been taken into account, e.g. culture media, antibiotics and disposable plasticware, unless they constitute a significant cost to the process.

[1] Invitrogen custom DNA oligos, 25 nmole desalted synthesis; £0.26 per base (ThermoFisher Scientific, UK)
[2] Phusion High-Fidelity DNA Polymerase; £78.75 for 100 units (ThermoFisher Scientific, UK)
[3] DpnI; £57.00 for 1000 units (NEB, UK)
[4] Zymo DNA Clean and Concentrator-5; £278.00 for 200 (Cambridge Bioscience)
[5] 5α Competent E. coli (High Efficiency); £166.00 for 20 x 0.05 mL (NEB, UK)
[6] TubeSeq Labels providing a ~1 kB read; €495.00 for 50 (Eurofins Genomics, Germany)
[7] Sterile Electroporation Cuvette, 2 mm gap width; £228.00 for 50 (Cole-Parmer, UK)
[8] QIAprep Spin Miniprep Kit; £68.60 for 50 (Qiagen, UK)
[9] High fidelity restriction enzymes, e.g. EcoRI & HindIII; £43.00 for 10000 units each (NEB, UK)
[10] Agarose, wide range for molecular biology; £77.30 for 10 g makes 20 gels of 50 mL (Sigma, UK)
[11] SYBR™ Safe DNA Gel Stain; £56.75 for 400 µL makes 80 gels of 50 mL at 1 in 10,000 dilution (ThermoFisher Scientific, UK)
[12] HyperLadder™ 1kb; £31.00 for 100 lanes (Bioline, UK)
[13] Zymo Gel DNA Recovery Kit; £288.00 for 200 (Cambridge Biosciences)
[14] T4 DNA ligase; £56.00 for 20000 units (NEB, UK)
[15] REDTaq® ReadyMix™ PCR Reaction Mix; £37.10 for 50 (Sigma, UK)
[16] PmeI; £60.00 for 500 units (NEB, UK)

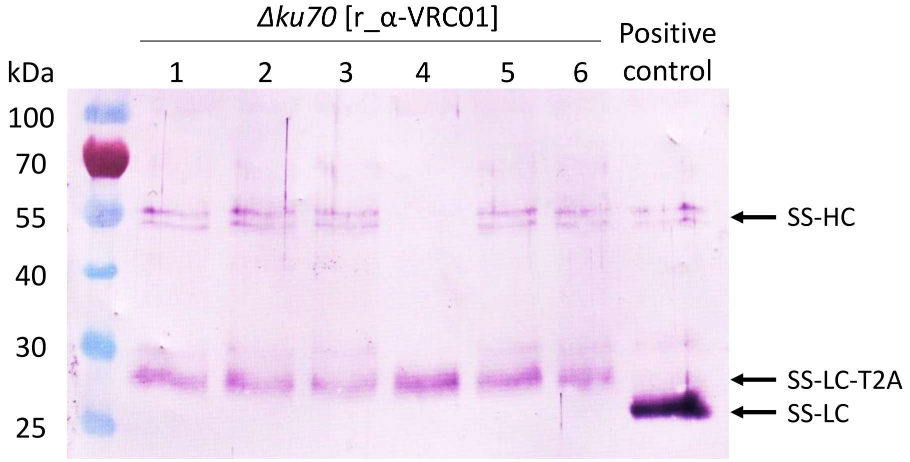

Figure S1 – Expression of the monoclonal antibody VRC01 from *Pichia pastoris Δku70* using a bicistronic vector, where the light (LC) and heavy chains (HC) are separated by the T2A self-processing peptide from *Thosea asigna*. Both LC and HC were expressed with the a murine IgG1 N-terminal secretion signal (SS). Six clones were cultivated in a 24 well plate at 20°C for 72 hours, feeding with 0.5% methanol every 24 hours. Expression was detected by 12% SDS-PAGE and Western blot with an alkaline phosphatase-conjugated rabbit anti-human IgG which detects both heavy and light chains. Positive control: VRC01 expression at 2.595 µg mL^{-1} from *Pichia pastoris Δku70*, where the light and heavy chains were expressed from separate, chromosomally integrated plasmids in 24 well plates at 20°C for 72 hours. Sample provided by Rochelle Aw, with a concentration determined by Paul McKay using ELISA detection of total immunoglobulin [1]. The full fusion protein has a predicted weight of 78.5 kDa; with the two polypeptides SS-LC-T2A and SS-HC weighing 26.7 and 51.9 kDa, respectively. The increase in size in light chain in the test expression compared to the control is a result of the T2A peptide (EGRGSLLTCGDVEENPGP); as translation is terminated at the final proline of the sequence, the light chain polypeptide is expressed with the C-terminal T2A sequence [2] calculated as an additional 1.8 kDa. The doublet band observed at around 55 kDa may be a consequence of differential processing of the N-terminal murine secretion signal; the absence of this in clone 4 is indicative of strain instability.

References

1. Aw, R., McKay, P. F., Shattock, R. J. & Polizzi, K. M. Expressing anti-HIV VRC01 antibody using the murine IgG1 secretion signal in *Pichia pastoris*. *AMB Express* **7**, 70 (2017).
2. Geier, M., Fauland, P., Vogl, T. & Glieder, A. Compact multi-enzyme pathways in *P. pastoris*. *Chem. Commun. (Camb.)* **51**, 1643-1646 (2015).
